# Novel Stepped Care Approach to Provide Education and Exercise Therapy for Patellofemoral Pain: Feasibility Study

**DOI:** 10.2196/18584

**Published:** 2020-07-22

**Authors:** Danilo De Oliveira Silva, Marcella F Pazzinatto, Kay M Crossley, Fabio M Azevedo, Christian J Barton

**Affiliations:** 1 La Trobe Sport and Exercise Medicine Research Centre School of Allied Health, Human Services and Sport La Trobe University Melbourne Australia; 2 Laboratory of Biomechanics and Motor Control Sao Paulo State University Presidente Prudente Brazil

**Keywords:** pain, internet, knee, rehabilitation, eHealth

## Abstract

**Background:**

Patellofemoral pain (PFP) impairs joint- and health-related quality of life and may be associated with knee osteoarthritis. We developed a novel, 2-phase, stepped-care approach for PFP, combining (1) self-directed web-based education and exercise therapy with (2) physiotherapist-supported education and exercise therapy. Physiotherapy sessions can be provided using 2 different modalities: face-to-face and telerehabilitation.

**Objective:**

This study aims to (1) determine the feasibility of our stepped-care approach, (2) explore patient-reported outcomes following self-directed web-based education and exercise therapy in people with PFP (phase 1), and (3) estimate the differences in treatment effects between face-to-face and telerehabilitation to support further education and exercise therapy (phase 2) in those who had not completely recovered following self-directed care.

**Methods:**

Phase 1 involved 6 weeks of self-directed web-based education and exercise therapy. Phase 2 involved random allocation to a further 12 weeks of physiotherapist-led (up to 8 sessions) education and exercise therapy delivered face-to-face or via telerehabilitation to participants who did not rate themselves as completely recovered following phase 1. Feasibility indicators of process, adherence, and participant retention were collected as primary outcomes alongside patient-reported outcomes on Global Rating of Change and knee pain, disability, knee-related quality of life, pain catastrophism, kinesiophobia, and knee self-efficacy. All participants were assessed at baseline, 6 weeks, and 18 weeks.

**Results:**

A total of 71 participants were screened to identify 35 participants with PFP to enter the study. Overall, 100% (35/35) and 88% (31/35) of the participants were followed up with at 6 and 18 weeks, respectively. In phase 1 of the study, participants accessed the My Knee Cap website for an average of 6 (7.5) days and performed the exercises for an average of 2.5 (3.6) times per week. A total of 20% (7/35) of the participants reported that they had completely recovered at 6 weeks. Furthermore, 93% (26/28) of the participants who were followed up and had not completely recovered at 6 weeks agreed to be enrolled in phase 2. No statistically significant differences were found between the face-to-face and telerehabilitation groups for any outcome. The novel stepped-care approach was associated with marked improvement or complete recovery in 40% (14/35) of the participants following phase 1 and 71% (25/35) of the participants following phase 2.

**Conclusions:**

Self-directed web-based education and exercise therapy for people with PFP is feasible, as noted by the high rate of participant retention and home exercise adherence achieved in this study. Furthermore, 20% (7/35) of people reported complete recovery at 6 weeks. Both face-to-face and telerehabilitation physiotherapy should be considered for those continuing to seek care, as there is no difference in outcomes between these delivery modes. Determining the efficacy of the stepped-care model may help guide more efficient health care for PFP.

## Introduction

### Background

Patellofemoral pain (PFP) is the most common knee condition experienced by young adults, affecting approximately one in four people worldwide [1]. PFP is characterized by diffuse anterior knee pain during activities that load the patellofemoral joint, including stair negotiation, running, and squatting [2,3]. PFP also impairs joint- and health-related quality of life [4]; reduces physical function [2]; results in fear of movement [5]; alters movement patterns [6,7]; and is associated with manifestations of peripheral and central sensitization, including lower pressure pain threshold and facilitated temporal summation of pain [8].

Exercise therapy is considered the cornerstone treatment for PFP [9-11]. When provided with or without other treatments (manual therapy, taping, and bracing), exercise therapy reduces pain, improves function, and leads to greater recovery rates in the short- and long-term compared with a placebo or *wait and see* approach (when participants do not receive any intervention over the study period) [9,12,13]. However, 57% of people with PFP report unfavorable outcomes 5 to 8 years following treatment [14]. Patient education for PFP is considered a key treatment by international experts to optimize self-management and long-term outcomes [9,11,15]. Our recent systematic review indicates that patient education may lead to similar short-term outcomes for pain and function as exercise therapy in PFP, but it is under researched [16]. During the development of an education leaflet for people with PFP, patients consistently requested a dedicated website to facilitate patient education [17]. Web-based interventions might facilitate self-directed education and exercise therapy to reduce health care–related costs at the system and personal levels [18]. Self-directed web-based interventions for low back pain [19,20] and osteoarthritis [21] can lead to improved patient-reported outcomes, including disability and perceived benefit compared with the *wait and see* approach. The impact of self-directed web-based interventions for people with PFP is unknown.

Implementation of a stepped-care model for PFP could benefit both patients and health systems. Stepped care can be defined as a staged evidence-based system comprising hierarchically delivered interventions, from the least to the most intensive, linked to patients’ needs [22,23]. The goal is to provide effective care with the least intensive treatment. Self-directed web-based care may reduce the burden of health professional consultations, but it is unlikely to benefit or satisfy all people with PFP. Additional supported education and exercise therapy may provide better outcomes in those continuing to seek care because of persistent pain. Education and exercise therapy can be provided via face-to-face or telerehabilitation delivery. Telerehabilitation produces similar benefits as the face-to-face delivery across several chronic conditions [19,24,25], including reports of no difference in pain and function when compared with face-to-face rehabilitation following a total knee replacement [26]. No study has compared telerehabilitation with face-to-face delivery of physiotherapy care in people with PFP.

### Objectives

This study was designed to (1) evaluate the feasibility of a stepped-care approach for people with PFP, (2) explore the effect of self-directed web-based education and exercise therapy on the perceived recovery and clinical outcomes of people with PFP (phase 1), and (3) estimate the differences in treatment effects between face-to-face and telerehabilitation delivery of physiotherapy to support further education and exercise therapy (phase 2) in those who did not completely recover following initial self-directed care.

## Methods

### Reporting, Registry, and Ethics Approval

This study was reported in accordance with the Consolidated Standards of Reporting Trials statement [27]. The study was approved by the La Trobe University Human Ethics Committee (process number: HEC17-102), and all participants provided written and verbal informed consent. We used 2 separate consent forms for phases 1 and 2 of our study. The protocol was a priori registered and approved by the Australian New Zealand Clinical Trials Registry (ACTRN12618000224224).

### Participants

Participants with PFP aged between 18 and 40 years were recruited using advertisements at La Trobe University and gyms of Melbourne (Australia) and on social media (Facebook, blogs, and Twitter) between February 26 and July 1, 2018.

Eligibility criteria were based on a consensus statement on terminology, definitions, and clinical examination of people with PFP [2]. The following eligibility criteria were assessed by an experienced (>6 years) physiotherapist from the research group. Participants were included if they presented anterior or retropatellar pain (1) corresponding to at least thirty on a 100-mm visual analog scale (VAS) in the previous week; (2) for at least 3 months; or (3) during at least two or more activities from prolonged sitting, squatting, kneeling, running, ascending and descending stairs, jumping, and landing. Exclusion criteria included a history of any lower limb surgery, history of patellar subluxation or dislocation, ligament or meniscus tears assessed clinically, presence of neurological diseases, or individuals who had received oral steroids and opiate treatment in the last month [28].

### Procedures

This study was designed to evaluate the feasibility of a stepped-care approach, including a randomized, clinical, single-center trial, comparing physiotherapy delivery modes. The novel stepped-care approach evaluated consists of 2 phases.

Phase 1 involved a pre-post design where all participants received 6 weeks of self-directed web-based education and exercise therapy.

Phase 2 involved a parallel-group randomized clinical trial (RCT). Participants were not informed of the existence of phase 2 when entering phase 1. Participants who were offered inclusion in phase 2 were those who did not rate themselves as *completely recovered* on the Global Rating of Change (GROC) 6-item Likert scale following phase 1. Each patient was offered the option to receive further 12 weeks of physiotherapy, which involved either face-to-face or telerehabilitation delivery, allocated randomly. All participants were reassessed after phase 2 (ie, at 18 weeks). Participants not receiving treatment during phase 2 (ie, those who *completely recovered* after 6 weeks) were also followed up at 18 weeks.

### Randomization and Blinding in Phase 2

A member of the research team not involved in data collection generated randomization lists (block randomization; block size of 4-6) using a random number generator on the website (sealedenvelope.com). Group allocations were concealed using sequentially numbered, sealed opaque envelopes, which were opened by one member of the research team not involved in data collection or randomization following baseline assessment.

#### Assessor

The assessor was blinded to participant group allocation.

#### Participants

Participants were told that they would be randomly allocated to one of the two different education and exercise therapy treatments. Once randomized, care was taken to ensure that the participants were unaware of the details of the alternative program (delivery mode). Participants were also instructed not to reveal the details of their allocated intervention to the blinded outcome assessor at the final follow-up (18 weeks).

#### Therapist

A total of 5 registered physiotherapists (>5 years of clinical experience) delivered the physiotherapy treatment—3 physiotherapists delivered the intervention in face-to-face mode and 2 physiotherapists delivered telerehabilitation via Skype. Precautions were taken to ensure that the physiotherapists were unaware of the existence of another intervention group, with physiotherapists believing that they were participating in a case series design to evaluate the feasibility of the respective intervention they delivered.

### Outcomes

**Figure 1:**
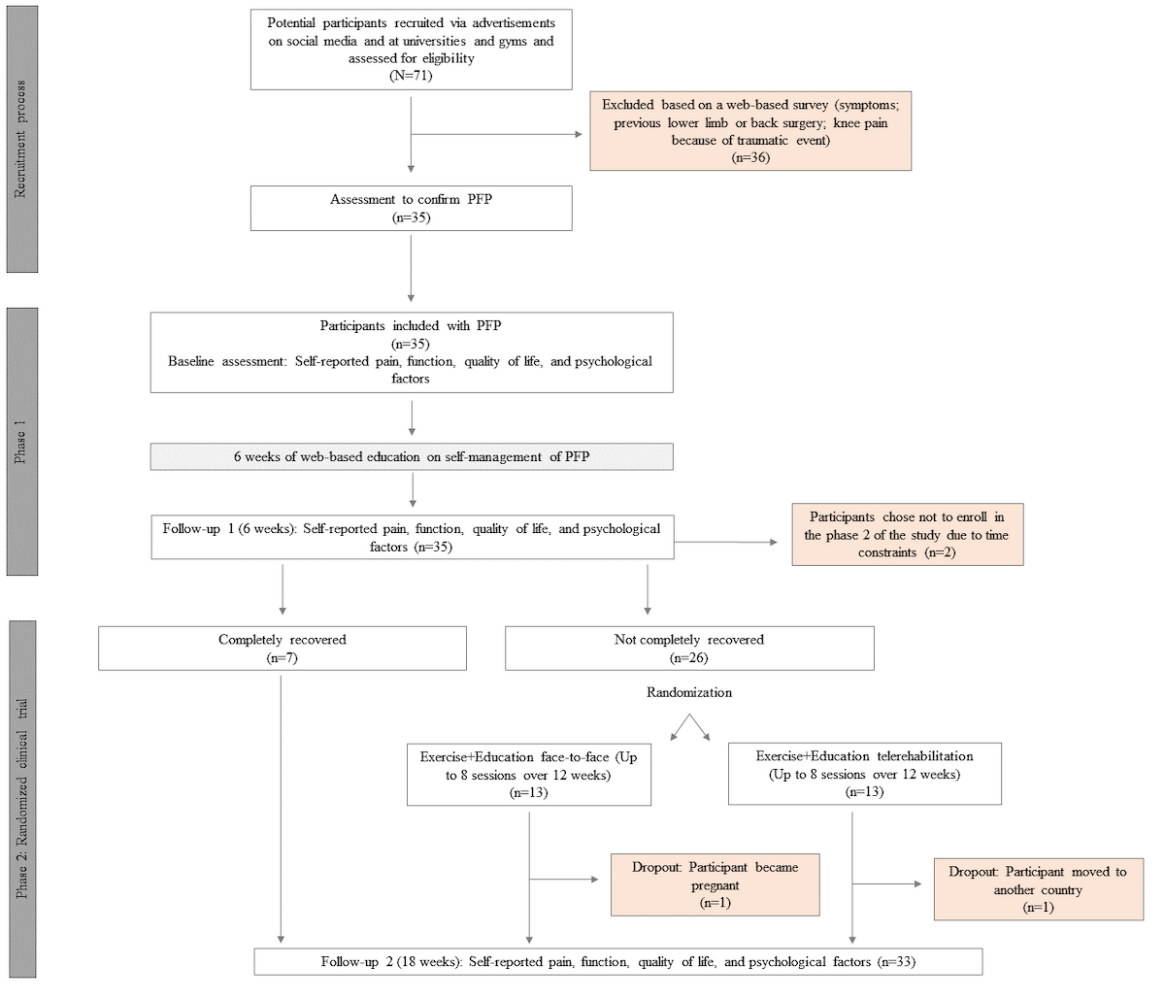
Flow chart of the study. PFP: patellofemoral pain.

The baseline, 6-week, and 18-week follow-up face-to-face assessments (Figure 1) were performed by the same blinded assessor at La Trobe Sports and Exercise Medicine Research Centre, La Trobe University, Melbourne, Australia.

All participants were asked to attend one 60-min initial screening and assessment at the university’s physiotherapy department. All questionnaires were self-administered, and participants completed the questionnaires independently on paper. Before starting the questionnaires, participants were asked to report their age in years. Body mass and height were measured using a scale and a measuring tape fixed on the wall, respectively. BMI was then calculated as weight in kilograms divided by height in meters squared. Participants also reported their symptom duration (months).

### Primary Outcome

Recruitment rates were recorded and defined as the number of participants recruited each week. The consent rate was calculated by dividing the number of people who met the inclusion criteria by the number of people who consented to participate in both phases 1 and 2.

Adherence to phase 1 was monitored by assessing the number of accesses to the *My Knee Cap* website, monitored through an algorithm embedded in the website. Participants also completed an exercise log daily for 6 weeks to indicate the days they performed the exercises available on the website. Adherence to phase 2 was monitored through the number of appointments scheduled with the physiotherapist for each group. In addition, all participants received a logbook to add information regarding how many times they accessed the website and performed the exercises each week and to record any adverse events (defined as negative consequences of care that result in unintended injury or illness that may or may not have been preventable).

### Primary Estimates of the Treatment Effect

#### GROC

Participants rated their perceived recovery at 6 weeks (phase 1) and 18 weeks (phase 2) following trial commencement on a 6-item Likert scale: (1) completely recovered, (2) markedly better, (3) moderately better, (4) same, (5) moderately worse, and (6) markedly worse. GROC is highly reliable, easily understandable, sensitive to change, and the preferred measure of pain and disability among people with PFP [29]. It also moderately correlates with pain and disability and strongly correlates with health-related quality of life changes [30].

#### Pain (VAS)

The worst knee pain intensity during the previous week was assessed on a 100-mm VAS, with 0 indicating *no pain* and 100 indicating *the worst pain possible* [31]. The minimal clinically important difference for people with PFP is 20 mm [32]. The VAS has been validated for people with PFP, and it has been reported to demonstrate high test-retest reliability [32].

### Secondary Estimates of the Treatment Effect

#### Disability

The Anterior Knee Pain Scale (AKPS) is a self-reported 13-item questionnaire, with scores ranging from 0 to 100 and higher values indicating better knee function [33]. The minimal clinically important difference for people with PFP is 10 points [32]. AKPS has been validated for people with PFP and has been reported to demonstrate high test-retest reliability [32].

#### Knee-Related Quality of Life

The Knee Injury and Osteoarthritis Outcome Score-Quality of Life subscale is a self-reported 4-item subscale with responses ranging from 0 to 4. Responses are transformed to a scale of 0 to 100, with 0 representing extreme knee problems and 100 representing no knee problems at all [34].

#### Pain Catastrophizing

The Pain Catastrophizing Scale (PCS) is a self-reported 13-item questionnaire to describe the various thoughts and feelings a person might experience when they are in pain. Scores range from 0 to 52, with higher values indicating more severe catastrophic thoughts about pain [35]. PCS has been previously validated for people with musculoskeletal conditions and has high test-retest reliability [36].

#### Kinesiophobia

The Tampa Scale for Kinesiophobia (TSK) is a self-reported 17-item questionnaire to quantify fear of movement. Scores range from 17 to 68, with higher values indicating greater fear [37,38]. TSK has been previously validated for people with musculoskeletal conditions and has moderate test-retest reliability [37].

#### Knee Self-Efficacy

The Knee Self-Efficacy Scale (K-SES) is a self-reported 22-item questionnaire where the participant rates the certainty about the capability of performing an activity, despite pain/discomfort, on an 11-point Likert scale, ranging from 0 (not at all certain) to 10 (very certain). The sum of the item scores is calculated and then divided by the number of items, yielding a total K-SES score ranging from 0 to 10. Higher scores indicate greater knee self-efficacy.

#### Adverse Events

Participants were given a specific form to record any adverse symptoms or events they experienced during the study (eg, pain flares, muscle soreness, and injury unrelated to the trial). No instructions were provided regarding the intensity of pain that would characterize an adverse event.

### Intervention

#### Phase 1 (0 to 6 Weeks)

During this phase, all participants received the same intervention: access to the *My Knee Cap* website for 6 weeks. The website provides education to participants with PFP on self-management of their knee pain based on current evidence [10,11,15], along with a self-directed exercise therapy program. The website was designed by the authors DS and CB, with the assistance of a web designer, to be both didactic and engaging, with a simple user interface and navigation appropriate for patients with low health literacy. We asked for feedback on the content, language, and functionality of the website from one layperson diagnosed with PFP (end user), one physiotherapist experienced in managing PFP, and one researcher with a strong track record of publications in PFP. Refinements were made to the website based on this feedback before the commencement of the study.

The website provides public access to 4 main sections: (1) understanding your pain, (2) treatment options, (3) exercise program, and (4) patient stories.

The *understanding your pain* section provides information covering a variety of subjects, including diagnosis, prognosis, incidence and prevalence, noisy knees, fear of movement, self-management of exercise load, and self-management of pain.

The *treatment options* section includes treatment options that can be beneficial (taping/bracing, foot orthosis, and exercises) and information about common treatments with inconsistent evidence (knee surgery, ultrasound, and platelet-rich plasma).

The *exercise program* section is focused on 4 types of exercises targeting the trunk, hip, and knee muscles, based primarily on a previously published exercise therapy trial. The participants had access to videos demonstrating the exercises proposed in the *exercise program* section. Explanations about how to progress the exercises were also provided (Multimedia Appendix 1).

The *patient stories* section presents the stories of 2 patients (1 woman and 1 man), from a private physiotherapy clinic in Melbourne, with knee pain who had successful outcomes after engaging in education and exercise therapy.

All information on the website was provided in plain language, with multimedia resources such as infographics, animated videos, and podcasts, to facilitate engagement and understanding. After the baseline assessment, a physiotherapist introduced the content of the website and gave the password of the exercise videos that were included on the website during a 30-min orientation session. In addition, participants were asked to complete one exercise of each type 3 times a week for a period of 6 weeks and access the website as often as needed.

#### Phase 2 (6 to 18 Weeks)

Participants who reported themselves to be *completely recovered* did not receive further treatment during phase 2. Participants who reported not being completely recovered were randomized to 12 weeks of face-to-face– or telerehabilitation-delivered education and exercise therapy with a physiotherapist.

Participants were permitted up to 8 sessions with their physiotherapists to receive guidance on education and exercise therapy. The upper limit of physiotherapy sessions was determined through a previous feasibility study [39], high-quality RCTs [13], and discussion with physiotherapists providing our intervention. To reflect clinical practice, we did not specify a minimum number of sessions and at what exact time points physiotherapy sessions should occur. Typically, physiotherapy sessions were closer together in the early stages (eg, every 1-2 weeks) and spread out toward the end of the 12 weeks (eg, monthly), as participants became more confident with their home exercise programs and knowledgeable about the condition. Education and exercise therapy that were provided were similar to what was available on the website, and the website was used to reinforce educational messages and exercise where relevant. The exercise therapy program and education content can be found on the *My Knee Cap* website and Multimedia Appendix 1. Physiotherapists were permitted to provide additional exercise input, including gym-focused progression, if deemed appropriate. When required, gym memberships were provided.

### Statistical Analyses

Statistical analyses were performed using SPSS version 23 (IBM, SPSS Inc). The level of significance was set a priori at *P*<.05. All statistical analyses were conducted by an investigator blinded to the group allocation.

#### Phase 1

Global perceived recovery after 6 weeks of self-directed web-based education and exercise therapy was reported descriptively (percentages). Within-group changes in primary and secondary estimates of effects were evaluated using paired *t* tests. Effect sizes (ESs) were calculated to guide the interpretation of the power of the comparison. ES values were defined as small (0.2-0.5), medium (0.51-0.80), or large (≥0.81) [40]. Correlations of exercise adherence with changes in patient-reported outcomes were performed using Pearson coefficient correlations.

#### Phase 2

Demographic data from the 2 groups (face-to-face and telerehabilitation) at 6 weeks were compared using independent *t* tests. Intention-to-treat analyses were used for all outcomes. We performed a 2×5 chi-square test for independence to investigate whether there was an association between GROC outcomes and the groups to which the participants were allocated at the end of 18 weeks (face-to-face and telerehabilitation groups). Independent *t* test and ES were used to compare patient-reported outcomes between the face-to-face and telerehabilitation groups. Using the same criteria as phase 1, ES values were defined as small (0.2-0.5), medium (0.51-0.80), or large (≥0.81) [40].

## Results

### Recruitment and Feasibility

Between February 2018 and July 2018, 35 participants (27 women and 8 men) with PFP were recruited from 71 potential candidates. The recruitment rate was 2 participants per week over an 18-week period, with a consenting rate of 100% (35/35). Overall, 77% (27/35) of the participants included were recruited via advertisements on social media, 11% (4/35) via advertisements at La Trobe University, and 11% (4/35) via advertisements at gyms in the neighborhood of La Trobe University. The trial was completed in December 2018, with 100% (35/35) of the participants followed up at 6 weeks and 88% (31/35) followed up at 18 weeks. For phase 2, the consenting rate was 93% (26/28), with 2 participants choosing not to enter because of time constraints, despite ongoing symptoms (Figure 1).

### Adherence

#### Phase 1

All participants accessed the website at least once, with web usage data indicating that the participants accessed the website for an average of 6 (7.5) days over the 6 weeks (Multimedia Appendix 2). Exercises were reported to be performed on an average of 15 (12.2) days over 6 weeks, equating to an average of 2.5 (3.6) times per week (18/35, 52% of the participants completed the exercises on an average of at least three times per week). Low to moderate correlations were found for exercise adherence (total number of days on which exercise was performed) with self-reported pain (*r*=0.33; *P*=.03), knee-related quality of life (*r*=−0.52; *P*=.001), self-reported function (*r*=−0.37; *P*=.01), pain catastrophizing (*r*=0.52; *P*=.001), kinesiophobia (*r*=0.39; *P*=.009), and knee self-efficacy (*r*=−0.51; *P*=.001).

#### Phase 2

Participants enrolled in phase 2 (Multimedia Appendix 3) performed an average of 4.5 (1.5) physiotherapy sessions in the face-to-face group and an average of 5.2 (2.1) physiotherapy sessions in the telerehabilitation group.

### Adverse Events

#### Phase 1

One participant reported an adverse event unrelated to the trial. Furthermore, 11 participants reported knee pain flares or muscle soreness of low intensity while performing the exercises, with most occurring in the first week (Multimedia Appendix 2) and no impact on participation in future exercise sessions.

#### Phase 2

Two adverse events unrelated to the trial were reported, one from each group. One participant fell on their knees while running to catch a bus, but this event did not alter the treatment. The other participant fell on a staircase, which led to a few scratches on her knee, delaying physiotherapy treatment for 2 weeks. No adverse event related to the trial was reported because of physiotherapy consultations or exercise therapy during phase 2.

### Secondary Outcomes

#### Phase 1

After 6 weeks of self-directed web-based education and exercise therapy, 20% (7/35) of the participants starting the trial reported that they had *completely recovered*, 20% (7/35) were markedly better, 40% (14/35) were moderately better, 17% (6/35) were same, and 3% (1/35) were moderately worse. Patient-reported outcomes at baseline and after 6 weeks are described in Table 1. Large improvements across the cohort occurred for worst knee pain in the previous week (ES=1.04), knee-related quality of life (ES=−1.13), disability (ES=−0.89), pain catastrophizing (ES=1.21), and knee self-efficacy (ES=−1.01), along with a medium improvement in kinesiophobia (ES=0.78).

**Table 1:** Comparisons between patient-reported outcomes at baseline and after the 6-week self-directed web-based education and exercise therapy.

Outcomes	At baseline, mean (SD)	At 6 weeks, mean (SD)	Mean difference (95% CI)
Worst knee pain (visual analog scale^a^, range: 0-100)	58.03 (17.54)	29.39 (25.52)	28.64 (18.88-38.39)
Knee-related quality of life (Knee Injury and Osteoarthritis Outcome Score-Quality of Life subscale^b^, range: 0-100)	41.86 (20.28)	66.67 (21.69)	−24.81 (−32.60 to −17.02)
Disability (Anterior Knee Pain Scale^b^, range: 0-100)	69.27 (13.41)	82.36 (13.87)	−13.09 (−18.34 to −7.85)
Pain catastrophizing (Pain Catastrophizing Scale^a^, range: 0-52)	21.73 (11.91)	10.09 (9.14)	11.64 (8.22-15.05)
Kinesiophobia (Tampa Scale for Kinesiophobia^a^, range: 17-68)	38.48 (6.14)	33.03 (6.83)	5.46 (2.99-7.92)
Knee self-efficacy (Knee Self-Efficacy Scale KSE-S^b^, range: 0-10)	5.57 (1.71)	7.28 (1.53)	−1.71 (−2.31 to −1.11)

^a^Higher scores indicate worse condition.

^b^Lower values indicate worse condition.

#### Phase 2

A total of 26 participants entered phase 2 and were randomly allocated to face-to-face– (n=13) or telerehabilitation-delivered education and exercise therapy (n=13). No between-group differences were found for demographics between these 2 groups (Table 2), and no between-group differences were found for any secondary estimates of treatment effect (Table 3). Both groups had one dropout during the intervention period, with one participant becoming pregnant and another moving to a different country.

**Figure 2:**
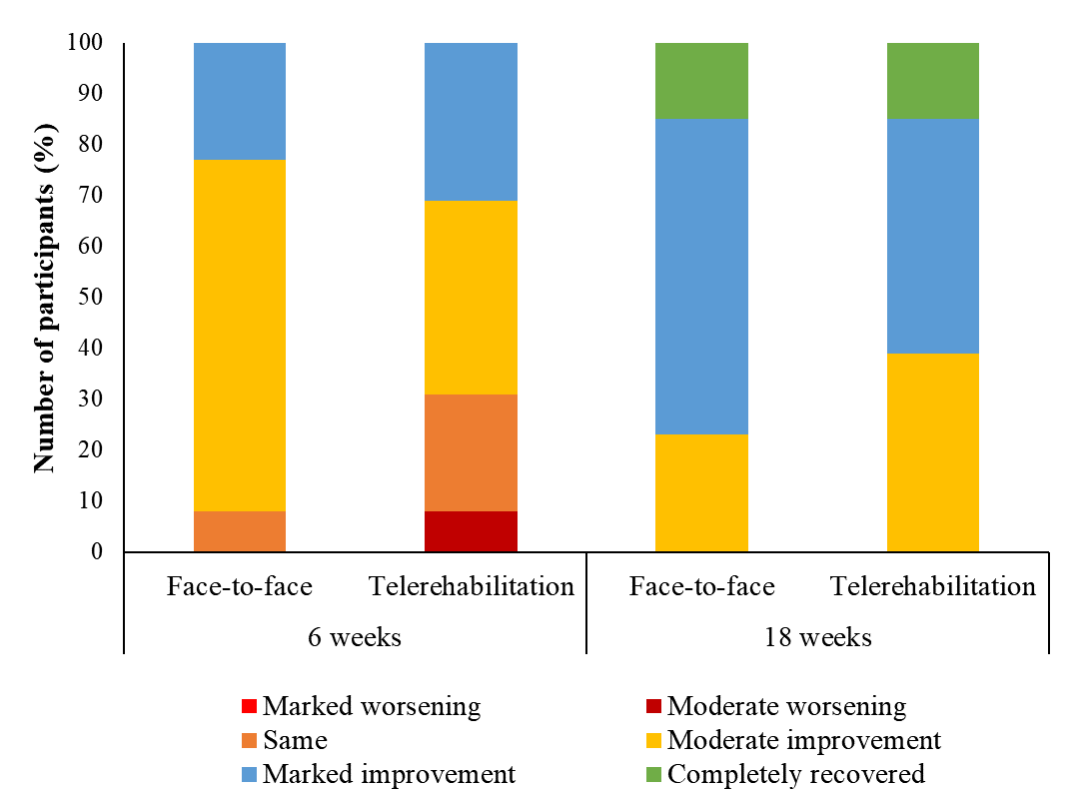
Global Rating of Change outcomes of the face-to-face and telerehabilitation groups.

The participants’ GROC outcomes after 18 weeks are shown in Figure 2. There was no significant difference in the proportion of GROC outcomes between the face-to-face and telerehabilitation groups (χ^2^_5_=2.03; *P*=.73).

No significant difference between face-to-face– and telerehabilitation-delivered education and exercise therapy was found for any patient-reported outcomes (Table 3). The between-group differences for the patient-reported outcomes had a moderate effect on worst knee pain in the previous week (ES=0.72) and a small effect on knee-related quality of life (ES=0.22), disability (ES=0.16), pain catastrophizing (ES=0.33), kinesiophobia (ES=0.22), and knee self-efficacy (ES=0.16). Overall, 71% (25/35) of all participants entering the study reported having completely recovered or markedly improved following phase 2.

**Table 2:** Demographic characteristics of face-to-face and telerehabilitation groups.

Variables	Face-to-face group, mean (SD)	Telerehabilitation group, mean (SD)	*P* value
Age (years)	32 (6)	31 (6)	.62
BMI (kg/m^2^)	27.58 (6.71)	26.46 (8.97)	.59
Symptoms duration (months)	53 (55)	32 (29)	.14

**Table 3:** Between-group comparisons for patient-reported outcomes at 6 and 18 weeks.

Outcomes		Face-to-face group, mean (SD)	Telerehabilitation group, mean (SD)	Mean difference (95% CI)
**Worst knee pain (visual analog scale, range: 0-100)**				
	6 weeks	32.31 (22.51)	42.31 (23.15)	−10.00 (−26.36 to 6.36)
	18 weeks	18.46 (18.46)	33.08 (22.13)	−14.62 (−31.25 to 2.02)
**Knee-related quality of life (Knee Injury and Osteoarthritis Outcome Score-Quality of Life subscale, range: 0-100)**				
	6 weeks	63.94 (16.76)	56.73 (21.87)	7.21 (−7.27 to 21.69)
	18 weeks	68.27 (21.57)	73.08 (21.71)	−4.81 (−20.56 to 10.95)
**Disability (Anterior Knee Pain Scale, range: 0-100)**				
	6 weeks	78.54 (12.61)	77.69 (12.94)	0.85 (−8.34 to 10.03)
	18 weeks	86.54 (10.59)	84.62 (13.49)	1.92 (−6.99 to 10.84)
**Pain catastrophizing (Pain Catastrophizing Scale, range: 0-52)**				
	6 weeks	8.23 (6.92)	14.46 (10.26)	−6.23 (−13.70 to 0.76)
	18 weeks	5.92 (6.02)	8.54 (9.44)	−2.62 (−8.29 to 3.06)
**Kinesiophobia (Tampa Scale for Kinesiophobia, range: 17-68)**				
	6 weeks	34.15 (7.72)	35.31 (5.01)	−1.15 (−6.07 to 3.76)
	18 weeks	30.38 (7.12)	31.77 (5.43)	−1.39 (−6.07 to 3.30)
**Knee self-efficacy (Knee Self-Efficacy Scale, range: 0-10)**				
	6 weeks	6.95 (0.93)	6.74 (1.75)	0.21 (−0.84 to 1.25)
	18 weeks	7.64 (1.45)	7.36 (1.98)	0.28 (−1.08 to 1.65)

## Discussion

### Summary of the Findings

This study confirms the feasibility of evaluating the efficacy of a stepped-care approach, including initial self-directed web-based education and exercise therapy, followed by physiotherapist-led education and exercise therapy. Overall, 35 participants with PFP were recruited from 71 potential candidates, with a recruitment rate of 2 participants per week and all eligible participants consenting to enter the trial. The follow-up rate was high for both phases of the trial, including 100% at 6 weeks and 88% at 18 weeks.

Six weeks of self-directed web-based education and exercise therapy was associated with 1 in 5 participants with PFP rating themselves as *completely recovered*. Self-directed care was associated with moderate to large improvements in pain, disability, kinesiophobia, pain catastrophizing, knee self-efficacy, and knee-related quality of life. Self-reported adherence to exercise (number of days completed) during phase 1 was related to clinical improvements in pain, function, and psychological outcomes. Although not significant, findings reported by Rathleff et al [41] in adolescents with PFP also indicate a link between greater exercise adherence and improved outcomes. In addition, a systematic review evaluating the influence of exercise dose on outcomes in knee osteoarthritis has reported that a greater number of exercise sessions per week is related to greater improvements in pain and function [42]. An interesting finding from our study is that participants reduced the number of exercise sessions they completed and the frequency in which they accessed the website during phase 1. Including consumers in the further development of the website may help to improve website engagement and exercise adherence.

Beneficial outcomes for pain and disability following self-directed web-based education and exercise therapy in this study are consistent with previous RCTs reporting improvements in the same outcomes following web-based interventions for low back pain [19] and hip and knee osteoarthritis [21,43]. Clinical improvements for the 20% (7/35) of participants who reported complete recovery at 6 weeks were sustained at 18 weeks, highlighting the potential for sustained recovery through self-directed care in people with PFP. Further research comparing self-directed web-based education and exercise therapy with a *wait and see* approach will help to determine efficacy and potential to reduce the current burden of overtreatment in health care systems around the world [44].

Overall, 4 out of 5 participants did not report complete recovery following self-directed web-based education and exercise therapy, indicating that some people may require or benefit from additional care. Face-to-face and telerehabilitation delivery of physiotherapy following initial self-directed care produced only small differences for all but one patient-reported outcome. The exception was for pain reduction, which was moderately greater (ES=0.72) in the face-to-face group, possibly because of differences exceeding 10 mm between groups at 6 weeks. Similar outcomes between face-to-face and telerehabilitation delivery of physiotherapy in this study are consistent with a large (n=201) high-quality noninferiority RCT in people following total knee joint replacement [26]. Thus, the physiotherapy delivery mode among people with knee pathologies may have minimal influence on patient-reported outcomes. Increasing the availability of physiotherapy care delivered via telerehabilitation is recommended to improve access to people with limited mobility, distressing symptoms, and/or inability to access center-based programs [18]. In addition, telerehabilitation approaches to physiotherapy practice could reduce the need for physiotherapy facilities requiring consultations and work-related travel for physiotherapists themselves.

Our findings indicate that 31% (11/35) of the participants reported low-intensity pain flares or muscle soreness while performing the exercises, with most occurring in the first week. This finding is not surprising, considering that participants need to initially learn new exercise skills, including how to manage and increase exercise loads without increasing knee pain. Reductions in the frequency of pain flares over the 6 weeks of self-directed care found in this study may be the result of participants improving their ability to independently manage exercise loads or ongoing active management leading to improved knee self-efficacy, which is typically impaired in people with PFP [45,46].

The overall proportion of participants reporting marked improvement or complete recovery across the cohort increased from 40% (14/35) at 6 weeks to 71% (25/35) at 18 weeks. High-quality RCTs, including long-term follow-up, are now needed to test this stepped-care approach against usual care or current best care [10] to evaluate efficacy, effectiveness, and cost-effectiveness. Findings from our study can be used to inform larger studies comparing face-to-face mode with telerehabilitation mode or investigating our stepped-care approach for people with PFP, which will allow more definitive conclusions.

### Limitations and Future Directions

Before making definitive recommendations regarding its potential value, our novel stepped-care approach requires comparison with a *wait and see* approach in both phases. Specifically, it is unclear whether self-directed care facilitated by our platform is superior to *wait and see* approach or whether any mode of physiotherapy following self-directed care can improve outcomes. Nonetheless, our findings suggest that if a self-directed web-based education and exercise therapy is provided as a first-line treatment, 1 in 5 patients may be able to independently manage their condition without the need for additional health professional referral.

Adherence seems to be related to better patient-reported outcomes. Future trials should develop strategies to optimize adherence to interventions (eg, digital support). In addition, the web-based platform was created with limited co-design processes [47]. Further modifications and improvements are currently being guided by additional qualitative research with consumers and physiotherapists and by using tools such as Health on the Net Foundation Code and DISCERN. Low computer and health literacy have been reported as barriers to improvement following other web-based interventions for chronic musculoskeletal conditions [48,49]. Our findings may not be applicable for people with low computer literacy skills. Further evaluation of efficacy, barriers, and cost-effectiveness of self-directed use of the website evaluated in this study, compared with usual care, is needed to inform potential implementation.

Although we measured the number of physiotherapy sessions attended during phase 2, we did not measure adherence to exercise, subjectively or objectively. Considering conflicting findings related to the importance of exercise adherence among people with knee pain [42,50], further evaluation of the importance of exercise adherence in PFP is strongly encouraged. In addition, approaches to treatment may have varied across the 5 physiotherapists providing care in our trial. However, all physiotherapists followed the same structure and content for education and exercise, guided by the *My Knee Cap* web-based platform. Each had a minimum of 5 years of clinical experience and received training and ongoing support as required, facilitated by an experienced physiotherapist (15 years) researcher involved in the study. Finally, we only included individuals with PFP aged between 18 and 40 years because of the high prevalence of PFP in this population [1], limiting the extrapolation of findings to adolescents and older adults with PFP.

### Conclusions

This study confirms the feasibility of evaluating the efficacy of a stepped-care approach, including initial self-directed web-based education and exercise therapy, followed by physiotherapist-led education and exercise therapy. Self-directed web-based education and exercise therapy were associated with 1 in 5 participants with PFP rating themselves as completely recovered at 6 weeks and having large improvements in pain. An additional 12 weeks of physiotherapy provided face-to-face or via telerehabilitation to support education and exercise therapy was associated with 71% (25/35) of participants reporting to be completely recovered or markedly improved. The absence of differences in outcomes between face-to-face and telerehabilitation delivery modes indicates that either mode could be considered depending on patient preference and need. Evaluating the efficacy of self-directed web-based education and exercise therapy as a stand-alone intervention and as part of a stepped-care model that includes additional physiotherapy care may help guide more efficient health care for people with PFP.

## Appendix

Multimedia Appendix 1 Exercise-therapy program.

Multimedia Appendix 2 Website usage and exercise adherence (phase 1).

Multimedia Appendix 3 Exercise adherence (phase 2).

## Abbreviations

AKPS: Anterior Knee Pain Scale
ES: effect size
GROC: Global Rating of Change
K-SES: Knee Self-Efficacy Scale
PCS: Pain Catastrophizing Scale
PFP: patellofemoral pain
RCT: randomized clinical trial
TSK: Tampa Scale for Kinesiophobia
VAS: visual analog scale
