# Supplementary material for: Novel Stepped Care Approach to Provide Education and Exercise Therapy for Patellofemoral Pain: Feasibility Study
Source: J Med Internet Res. 2020 Jul 22;22(7):e18584. doi: 10.2196/18584 (PMC7407256; doi:10.2196/18584)
Supplement: Multimedia Appendix 1 [file jmir_v22i7e18584_app1.pdf]

BRIDGES

Phase 1

Two Legged Bridging:

*How to progress exercise:**

- Option 1: increase speed, gradually increase speed without compromising technique

- Option 2: add weight, use weight on top of hips (i.e. plate at gym or large bag of rice) on top of hips

* You may choose either option to progress

Instructions:

- Hands over chest
- Tighten bottom muscles
- Tuck bottom under and lift hips up
- Don't arch your back at top of bridge
- Lower back down with bottom muscles

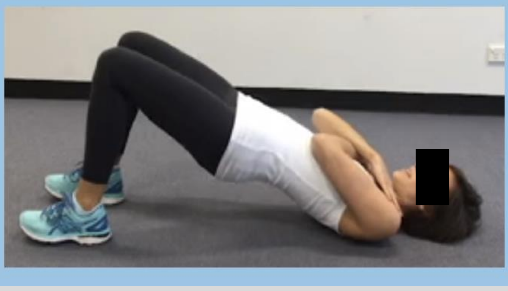

Dosage: 3 sets of 12 repetitions (60 seconds rest between sets)

Phase 2

One Leg Bridging:

*How to progress exercise:**

- Option 1: increase speed, gradually increase speed without compromising technique

- Option 2: add weight, use weight on top of hips (i.e. plate at gym or large bag of rice) on top of hips

* You may choose either option to progress

Instructions:

- Tighten bottom muscles
- Tuck bottom under and lift hips up
- Don't let hips rotate or drop
- Don't arch your back at top of bridge
- Lower back down with bottom muscles

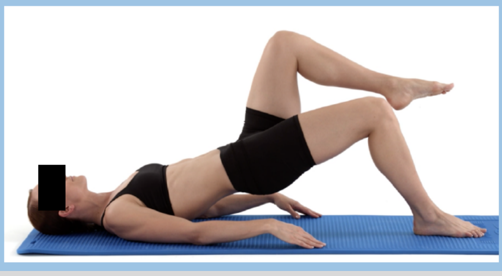

Dosage: 3 sets of 12 repetitions (60 seconds rest between sets)

CORE

Phase 1

Bench plank:

*How to progress exercise:**

- Option 1: Decrease incline to 30°

Instructions:

- Start with 45° incline
- Keep even weight through hands and feet
- Maintain a straight back and hips
- Should feel fatigue in core/abs area

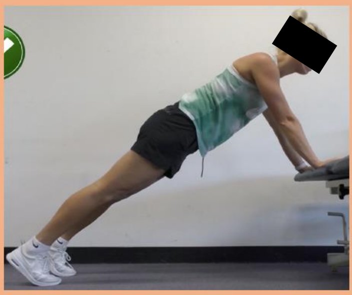

Dosage: 3 sets of 60 seconds hold (60 seconds rest between sets)

Phase 2

Elbow plank:

*How to progress exercise:**

- Option 1: Plank on hands
- Option 2: Bring one knee to chest

* You may choose either option to progress

Instructions:

- Start with 45° incline
- Keep even weight through hands and feet
- Maintain a straight back and hips
- Should feel fatigue in core/abs area

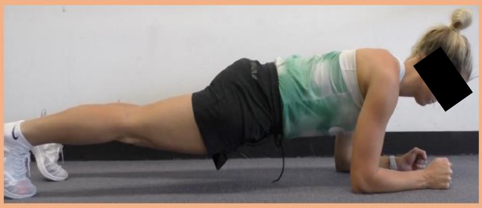

Dosage: 3 sets of 60 seconds hold (60 seconds rest between sets)

Phase 3

Side plank:

*How to progress exercise:**

- Option 1: Plank on legs straight on feet
- Option 2: Plank on hands and feet

* You may choose either option to progress

Instructions:

- Start with lying on side with knees on soft surface
- Keep hips straight in straight line with shoulders and knees
- Lift hips off the ground
- Make sure shoulders and back are straight and not rotated
- Tense bottom muscles
- Maintain straight posture
- Repeat on left and right side

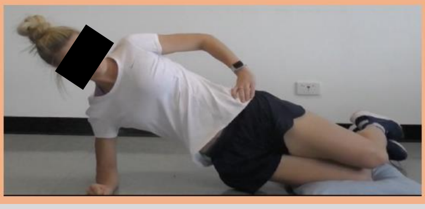

Dosage: 3 sets of 60 seconds hold (60 seconds rest between sets)

HIP ABDUCTORS

**Phase 1**	**Side leg lifts:**<br><br>*How to progress exercise:*<br><br>- Option 1: Add a resistance band, above knees or around ankles<br><br>- Option 2: Increase resistance band tension	**Instructions:**<br><br>- *Start lying on side without resistance*<br>- *Ensure gluteal activation and fatigue*<br>- *Abduct leg in line with body*<br>- *Avoid rotation of leg*<br><br>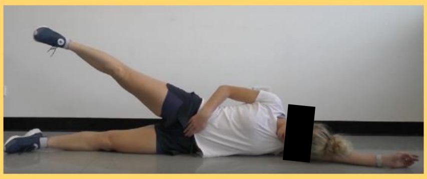<br>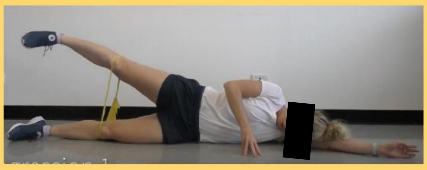
Dosage: 3 sets of 12 repetitions (rest 60 secs). Complete on left and right sides		

**Phase 2**	**Standing hip abduction:**<br><br>*How to progress exercise:*<br><br>- Option 1: Add resistance to band, by stepping away from resistance anchor or use a band with more resistance<br><br>- Option 2: Take hand off support	**Instructions:**<br><br>- *Fix resistance band to stable object*<br>- *Band/cable slightly above ankle*<br>- *Good spinal and hip control*<br>- *Ensure gluteal activation and fatigue*<br>- *Abduct leg in line with body*<br><br>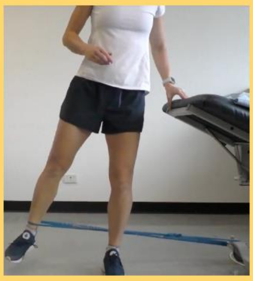
Dosage: 3 sets of 12 repetitions (rest 60 secs). Complete on left and right sides		

HIP ABDUCTORS

**Phase 3**	**Standing hip abduction:**<br><br>*How to progress exercise:*<br><br>- Option 1: Increase weight<br><br>- Home option: Do phase 2	**Instructions:**<br><br>*- Start in standing at weight you can do 12 times without compromising technique*<br>*- Maintain good spinal and hip control*<br>*- Ensure gluteal activation and fatigue*<br>*- Keep feet and legs pointing straight*<br><br>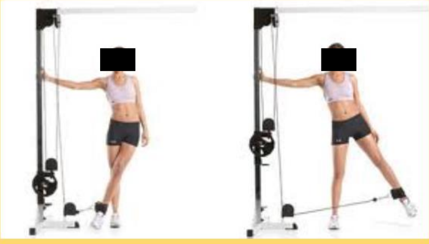
Dosage: 3 sets of 10 repetitions (rest 60 secs). Complete on left and right sides		

**Phase 4**	**Side stepping with band: Power based movement**<br><br>*How to progress exercise:*<br><br>- Option 1: Increase step distance when side stepping<br><br>- Option 2: Increase resistance of resistance band. Forward/diagonal progressions	**Instructions:**<br><br>*- Start with sidestepping with resistance that achieve fatigue 60 seconds*<br>*- Place band around ankles with feet pointed in*<br>*- Maintain good spinal and hip control trough exercise*<br>*- Step to side to place tension on band*<br>*- Keep feet and legs pointing straight*<br><br>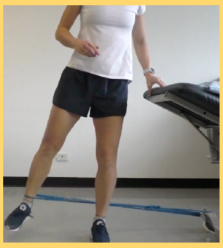
Dosage: 3 sets of 10 repetitions (rest 60 secs). Complete on left and right sides		

SQUATS

Phase 1

Wall sit:

How to progress exercise:

- Option 1: increase depth, start with a knee bend of 30°. Then gradually increase to 90° when ready
- Option 2: increase weight, start at 5kgs and gradually increase to 15kgs when ready

Instructions:

- *Start with a 30° knee bend*
- *Try and keep even pressure on both legs and feet*
- *Try and keep knees in line with your toes*
- *Don't bring knees forward past toes*
- *Keep shins vertical to the ground*

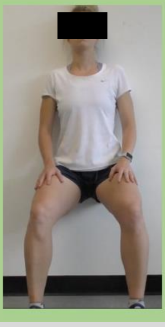

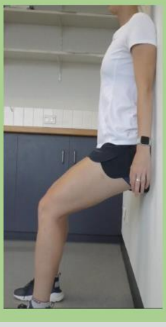

Dosage: 3 sets of 60 seconds hold (60 seconds rest between sets)

Phase 2

Two legged squats:

How to progress exercise:

- Option 1: increase depth, start with a squat to 30° knee bend. Then gradually increase to 90° when ready
- Option 2: increase weight, start with body weight
- Continue to increase weight as appropriate
- If you are unable to access a gym use weights/house hold heavy items at home and complete exercises

Instructions:

- *Stick bottom out, as though going to sit on edge of a chair*
- *Use a chair for feedback, but don't sit down on it*
- *Try and keep even pressure on both legs and feet*
- *Try and keep knees straight (knees over your toes)*

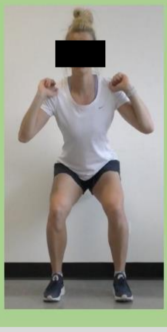

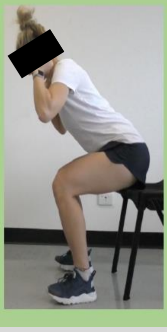

Dosage: 3 sets of 12 repetitions (60 seconds rest between sets)

Phase 3

One leg squats:

How to progress exercise:

- Option 1: increase depth, start with a squat to 30° knee bend. Then gradually increase to 90° when ready
- Option 2: increase weight, start at 5kgs and gradually increase to 15kgs when ready
- If you are unable to access a gym use weights/house hold heavy items at home and complete single leg exercises

Instructions:

- *Try and keep knee in line with your foot*
- *Don't drop or rotate hips*
- *Try and keep trunk / core straight without twisting or bending*
- *Keep even pressure on foot without lifting up heels or toes*
- *Complete for both left and right leg*

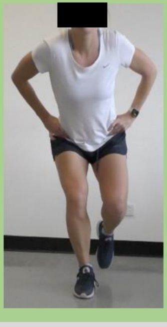

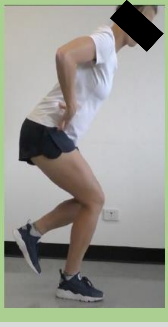

Dosage: 3 sets of 12 repetitions (60 seconds rest between sets)
