# Supplementary material for: Novel Stepped Care Approach to Provide Education and Exercise Therapy for Patellofemoral Pain: Feasibility Study
Source: J Med Internet Res. 2020 Jul 22;22(7):e18584. doi: 10.2196/18584 (PMC7407256; doi:10.2196/18584)
Supplement: Multimedia Appendix 2 [file jmir_v22i7e18584_app2.docx]

**Appendix 2:** Exercise log – Week 1

| **Participant ID** | **How many days did you use the website?** | **How many days did you do the exercises?** | **How many days have you had adverse event due to exercise?** | **Describe the adverse events** |
| --- | --- | --- | --- | --- |
| BEEP001 | 2 | 4 | 1 | Knee pain |
| BEEP002 | 3 | 4 | 0 | X |
| BEEP003 | 3 | 2 | 0 | X |
| BEEP004 | 4 | 3 | 1 | Muscle soreness |
| BEEP005 | 2 | 3 | 0 | X |
| BEEP006 | 4 | 1 | 0 | X |
| BEEP007 | 3 | 3 | 3 | Muscle soreness |
| BEEP008 | 3 | 4 | 2 | Knee pain |
| BEEP009 | 3 | 3 | 0 | X |
| BEEP010 | 2 | 3 | 6 | Knee pain |
| BEEP011 | 2 | 3 | 0 | X |
| BEEP012 | 1 | 2 | 0 | X |
| BEEP013 | 3 | 1 | 0 | X |
| BEEP014 | 5 | 2 | 2 | Knee pain |
| BEEP015 | 1 | 3 | 3 | Knee pain |
| BEEP016 | 4 | 4 | 0 | X |
| BEEP017 | 1 | 4 | 0 | X |
| BEEP018 | 5 | 5 | 3 | Knee pain |
| BEEP019 | 1 | 2 | 0 | X |
| BEEP020 | 2 | 5 | 0 | X |
| BEEP021 | 3 | 3 | 0 | X |
| BEEP022 | 4 | 3 | 0 | X |
| BEEP023 | 2 | 2 | 1 | Muscle soreness |
| BEEP024 | 1 | 2 | 0 | X |
| BEEP025 | 0 | 1 | 0 | X |
| BEEP026 | 4 | 3 | 2 | Muscle soreness |
| BEEP027 | 3 | 2 | 0 | X |
| BEEP028 | 3 | 5 | 0 | X |
| BEEP029 | 1 | 2 | 0 | X |
| BEEP030 | 1 | 3 | 1 | Knee pain |
| BEEP031 | 3 | 3 | 0 | X |
| BEEP032 | 5 | 2 | 0 | X |
| BEEP033 | 5 | 3 | 0 | X |
| BEEP034 | 3 | 3 | 0 | X |
| BEEP035 | 2 | 3 | 0 | X |
| **Mean (SD)** | **2.69 (1.33)** | **2.89 (1.04)** | **0.71 (1.32)** | **NA** |

Exercise log – Week 2

| **Participant ID** | **How many days did you use the website?** | **How many days did you do the exercises?** | **How many days have you had adverse event due to exercise?** | **Describe the adverse events** |
| --- | --- | --- | --- | --- |
| BEEP001 | 2 | 5 | 1 | Knee pain |
| BEEP002 | 2 | 5 | 0 | X |
| BEEP003 | 1 | 2 | 0 | X |
| BEEP004 | 2 | 3 | 0 | X |
| BEEP005 | 1 | 3 | 0 | X |
| BEEP006 | 0 | 0 | 0 | X |
| BEEP007 | 1 | 3 | 0 | X |
| BEEP008 | 0 | 3 | 1 | Knee pain |
| BEEP009 | 2 | 2 | 0 | X |
| BEEP010 | 1 | 4 | 0 | X |
| BEEP011 | 2 | 3 | 0 | X |
| BEEP012 | 0 | 3 | 0 | X |
| BEEP013 | 2 | 2 | 0 | X |
| BEEP014 | 1 | 3 | 1 | Knee pain |
| BEEP015 | 0 | 2 | 1 | Knee pain |
| BEEP016 | 1 | 3 | 0 | X |
| BEEP017 | 1 | 2 | 0 | X |
| BEEP018 | 1 | 5 | 2 | Knee pain |
| BEEP019 | 2 | 3 | 0 | X |
| BEEP020 | 3 | 4 | 0 | X |
| BEEP021 | 2 | 2 | 0 | X |
| BEEP022 | 2 | 2 | 0 | X |
| BEEP023 | 3 | 2 | 1 | Muscle soreness |
| BEEP024 | 1 | 2 | 0 | X |
| BEEP025 | 1 | 1 | 0 | X |
| BEEP026 | 3 | 3 | 2 | Muscle soreness |
| BEEP027 | 2 | 3 | 0 | X |
| BEEP028 | 3 | 6 | 0 | X |
| BEEP029 | 1 | 2 | 0 | X |
| BEEP030 | 0 | 3 | 1 | Knee pain |
| BEEP031 | 1 | 3 | 3 | Knee pain |
| BEEP032 | 1 | 4 | 2 | Knee pain |
| BEEP033 | 3 | 3 | 0 | X |
| BEEP034 | 2 | 4 | 0 | X |
| BEEP035 | 2 | 3 | 0 | X |
| **Mean (SD)** | **1.49 (0.91)** | **2.94 (1.17)** | **0.43 (0.77)** | **NA** |

Exercise log – Week 3

| **Participant ID** | **How many days did you use the website?** | **How many days did you do the exercises?** | **How many days have you had adverse event due to exercise?** | **Describe the adverse events** |
| --- | --- | --- | --- | --- |
| BEEP001 | 0 | 3 | 0 | X |
| BEEP002 | 1 | 2 | 0 | X |
| BEEP003 | 1 | 1 | 0 | X |
| BEEP004 | 0 | 2 | 0 | X |
| BEEP005 | 0 | 2 | 0 | X |
| BEEP006 | 0 | 0 | 0 | X |
| BEEP007 | 1 | 5 | 0 | X |
| BEEP008 | 2 | 3 | 1 | Knee pain |
| BEEP009 | 3 | 5 | 0 | X |
| BEEP010 | 1 | 4 | 1 | Knee pain |
| BEEP011 | 2 | 2 | 0 | X |
| BEEP012 | 0 | 0 | 0 | X |
| BEEP013 | 2 | 2 | 0 | X |
| BEEP014 | 0 | 3 | 0 | X |
| BEEP015 | 0 | 5 | 1 | Knee pain |
| BEEP016 | 2 | 3 | 0 | X |
| BEEP017 | 1 | 0 | 0 | X |
| BEEP018 | 0 | 7 | 0 | X |
| BEEP019 | 0 | 5 | 0 | X |
| BEEP020 | 1 | 4 | 0 | X |
| BEEP021 | 3 | 5 | 0 | X |
| BEEP022 | 2 | 3 | 0 | X |
| BEEP023 | 2 | 2 | 0 | X |
| BEEP024 | 1 | 1 | 0 | X |
| BEEP025 | 0 | 0 | 0 | X |
| BEEP026 | 4 | 4 | 0 | X |
| BEEP027 | 2 | 1 | 0 | X |
| BEEP028 | 0 | 5 | 0 | X |
| BEEP029 | 1 | 1 | 0 | X |
| BEEP030 | 0 | 2 | 0 | X |
| BEEP031 | 2 | 3 | 2 | Knee pain |
| BEEP032 | 0 | 4 | 0 | X |
| BEEP033 | 2 | 3 | 0 | X |
| BEEP034 | 2 | 4 | 0 | X |
| BEEP035 | 1 | 2 | 0 | X |
| **Mean (SD)** | **1.11 (1.06)** | **2.80 (1.72)** | **0.14 (0.42)** | **NA** |

Exercise log – Week 4

| **Participant ID** | **How many days did you use the website?** | **How many days did you do the exercises?** | **How many days have you had adverse event due to exercise?** | **Describe the adverse events** |
| --- | --- | --- | --- | --- |
| BEEP001 | 0 | 5 | 0 | X |
| BEEP002 | 1 | 3 | 0 | X |
| BEEP003 | 1 | 3 | 0 | X |
| BEEP004 | 0 | 3 | 0 | X |
| BEEP005 | 0 | 3 | 0 | X |
| BEEP006 | 0 | 0 | 0 | X |
| BEEP007 | 1 | 4 | 0 | X |
| BEEP008 | 4 | 4 | 1 | Knee pain |
| BEEP009 | 0 | 0 | 0 | X |
| BEEP010 | 0 | 3 | 0 | X |
| BEEP011 | 0 | 0 | 0 | X |
| BEEP012 | 0 | 0 | 0 | X |
| BEEP013 | 1 | 3 | 0 | X |
| BEEP014 | 0 | 1 | 0 | X |
| BEEP015 | 0 | 5 | 0 | X |
| BEEP016 | 1 | 4 | 0 | X |
| BEEP017 | 0 | 1 | 1 | Knee pain |
| BEEP018 | 0 | 5 | 0 | X |
| BEEP019 | 2 | 3 | 0 | X |
| BEEP020 | 2 | 3 | 0 | X |
| BEEP021 | 0 | 0 | 0 | X |
| BEEP022 | 2 | 3 | 0 | X |
| BEEP023 | 0 | 0 | 0 | X |
| BEEP024 | 0 | 0 | 0 | X |
| BEEP025 | 0 | 0 | 0 | X |
| BEEP026 | 3 | 3 | 2 | Muscle soreness |
| BEEP027 | 1 | 3 | 0 | X |
| BEEP028 | 0 | 4 | 0 | X |
| BEEP029 | 0 | 0 | 0 | X |
| BEEP030 | 0 | 3 | 3 | Knee pain |
| BEEP031 | 1 | 3 | 0 | X |
| BEEP032 | 0 | 4 | 0 | X |
| BEEP033 | 1 | 3 | 0 | X |
| BEEP034 | 0 | 0 | 0 | X |
| BEEP035 | 3 | 3 | 0 | X |
| **Mean (SD)** | **0.69 (1.04)** | **2.34 (1.69)** | **0.20 (0.62)** | **NA** |

Exercise log – Week 5

| **Participant ID** | **How many days did you use the website?** | **How many days did you do the exercises?** | **How many days have you had adverse event due to exercise?** | **Describe the adverse events** |
| --- | --- | --- | --- | --- |
| BEEP001 | 0 | 5 | 0 | X |
| BEEP002 | 0 | 2 | 0 | X |
| BEEP003 | 0 | 0 | 0 | X |
| BEEP004 | 0 | 3 | 0 | X |
| BEEP005 | 0 | 2 | 0 | X |
| BEEP006 | 0 | 0 | 0 | X |
| BEEP007 | 1 | 3 | 0 | X |
| BEEP008 | 1 | 1 | 0 | X |
| BEEP009 | 0 | 0 | 0 | X |
| BEEP010 | 0 | 2 | 0 | X |
| BEEP011 | 0 | 0 | 0 | X |
| BEEP012 | 2 | 0 | 0 | X |
| BEEP013 | 0 | 2 | 0 | X |
| BEEP014 | 0 | 2 | 0 | X |
| BEEP015 | 0 | 4 | 0 | X |
| BEEP016 | 1 | 5 | 0 | X |
| BEEP017 | 0 | 2 | 0 | X |
| BEEP018 | 1 | 5 | 0 | X |
| BEEP019 | 0 | 4 | 0 | X |
| BEEP020 | 0 | 4 | 0 | X |
| BEEP021 | 0 | 0 | 0 | X |
| BEEP022 | 3 | 3 | 0 | X |
| BEEP023 | 3 | 3 | 0 | X |
| BEEP024 | 0 | 0 | 0 | X |
| BEEP025 | 0 | 0 | 0 | X |
| BEEP026 | 3 | 3 | 0 | X |
| BEEP027 | 0 | 1 | 0 | X |
| BEEP028 | 0 | 1 | 0 | X |
| BEEP029 | 0 | 0 | 0 | X |
| BEEP030 | 0 | 2 | 0 | X |
| BEEP031 | 2 | 3 | 0 | X |
| BEEP032 | 0 | 4 | 0 | X |
| BEEP033 | 0 | 3 | 0 | X |
| BEEP034 | 1 | 1 | 0 | X |
| BEEP035 | 2 | 3 | 0 | X |
| **Mean (SD)** | **0.57 (0.96)** | **2.09 (1.61)** | **0.00 (0.00)** | **NA** |

Exercise log – Week 6

| **Participant ID** | **How many days did you use the website?** | **How many days did you do the exercises?** | **How many days have you had adverse event due to exercise?** | **Describe the adverse events** |
| --- | --- | --- | --- | --- |
| BEEP001 | 0 | 6 | 0 | X |
| BEEP002 | 0 | 3 | 0 | X |
| BEEP003 | 0 | 2 | 0 | X |
| BEEP004 | 0 | 3 | 0 | X |
| BEEP005 | 0 | 1 | 0 | X |
| BEEP006 | 0 | 1 | 0 | X |
| BEEP007 | 0 | 4 | 0 | X |
| BEEP008 | 1 | 3 | 0 | X |
| BEEP009 | 0 | 0 | 0 | X |
| BEEP010 | 0 | 3 | 0 | X |
| BEEP011 | 0 | 0 | 0 | X |
| BEEP012 | 0 | 0 | 0 | X |
| BEEP013 | 1 | 2 | 0 | X |
| BEEP014 | 0 | 3 | 0 | X |
| BEEP015 | 0 | 5 | 0 | X |
| BEEP016 | 3 | 3 | 0 | X |
| BEEP017 | 1 | 2 | 1 | Muscle soreness |
| BEEP018 | 4 | 4 | 0 | X |
| BEEP019 | 2 | 3 | 0 | X |
| BEEP020 | 1 | 4 | 0 | X |
| BEEP021 | 0 | 0 | 0 | X |
| BEEP022 | 2 | 2 | 0 | X |
| BEEP023 | 0 | 0 | 0 | X |
| BEEP024 | 0 | 0 | 0 | X |
| BEEP025 | 0 | 0 | 0 | X |
| BEEP026 | 3 | 3 | 0 | X |
| BEEP027 | 0 | 2 | 0 | X |
| BEEP028 | 0 | 1 | 0 | X |
| BEEP029 | 0 | 0 | 0 | X |
| BEEP030 | 0 | 0 | 0 | X |
| BEEP031 | 1 | 4 | 1 | Knee pain |
| BEEP032 | 0 | 5 | 1 | Knee pain |
| BEEP033 | 0 | 5 | 0 | X |
| BEEP034 | 2 | 3 | 0 | X |
| BEEP035 | 1 | 1 | 0 | X |
| **Mean (SD)** | **0.63 (1.04)** | **2.23 (1.74)** | **0.09 (0.28)** | **NA** |
