# Supplementary material for: Novel Stepped Care Approach to Provide Education and Exercise Therapy for Patellofemoral Pain: Feasibility Study
Source: J Med Internet Res. 2020 Jul 22;22(7):e18584. doi: 10.2196/18584 (PMC7407256; doi:10.2196/18584)
Supplement: Multimedia Appendix 3 [file jmir_v22i7e18584_app3.docx]

**Appendix 3**

Adherence of the face-to-face group to treatment

| **Participant ID** | **Group** | **Number of sessions** |
| --- | --- | --- |
| BEEP002 | Face-to-face | 5 |
| BEEP007 | Face-to-face | Withdraw |
| BEEP011 | Face-to-face | 3 |
| BEEP013 | Face-to-face | 4 |
| BEEP014 | Face-to-face | 5 |
| BEEP016 | Face-to-face | 3 |
| BEEP018 | Face-to-face | 6 |
| BEEP021 | Face-to-face | 3 |
| BEEP023 | Face-to-face | 6 |
| BEEP025 | Face-to-face | 3 |
| BEEP026 | Face-to-face | 6 |
| BEEP027 | Face-to-face | 5 |
| BEEP030 | Face-to-face | 6 |
| Total = 13 |  | Mean = 4.5 sessions |

Adherence of the online group to treatment

| **Participant ID** | **Group** | **Number of sessions** |
| --- | --- | --- |
| BEEP003 | Online | 6 |
| BEEP004 | Online | 6 |
| BEEP005 | Online | Withdraw |
| BEEP006 | Online | 4 |
| BEEP008 | Online | 6 |
| BEEP009 | Online | 4 |
| BEEP012 | Online | 6 |
| BEEP017 | Online | 4 |
| BEEP022 | Online | 3 |
| BEEP024 | Online | 3 |
| BEEP029 | Online | 8 |
| BEEP031 | Online | 6 |
| BEEP032 | Online | 7 |
| Total = 13 |  | Mean = 5.2 sessions |
